# Aberrant mitochondrial hsp60 expression affects mitochondria homeostasis and results in muscle dystrophy and premature death

**DOI:** 10.1038/s41419-025-08260-1

**Published:** 2026-01-08

**Authors:** Tsung-Hsien Chen, Chu-Kuang Chou, Kurt Ming-Chao Lin

**Affiliations:** 1 Institute of Biomedical Engineering and Nanomedicine, National Health Research Institutes, Zhunan, Taiwan; 2 Department of Internal Medicine, Ditmanson Medical Foundation Chia-Yi Christian Hospital, Chiayi, Taiwan; 3 Division of Gastroenterology and Hepatology, Department of Internal Medicine, Ditmanson Medical Foundation Chia-Yi Christian Hospital, Chiayi, Taiwan; 4 Obesity Center, Ditmanson Medical Foundation Chia-Yi Christian Hospital, Chia-Yi, Taiwan

**Keywords:** Chaperones, Mechanisms of disease

## Abstract

Heat shock protein 60 (HSP60) plays a vital role in maintaining mitochondrial homeostasis and essential functions and requires ATP for its assembly into chaperone complexes. This study aimed to investigate the long-term effects of HSP60 induction on mitochondrial homeostasis at varying doses and durations using HSP60 transgenic mice. In this study, we generated transgenic mice with elevated levels of native HSP60 using the LoxP-Cre system. These mice exhibited impaired postnatal development, skeletal muscle dystrophy, and increased mortality. Initially, excess HSP60 enhanced the mitochondrial oxidative respiratory capacity, which was later compensated for by increased glycolysis. Surplus HSP60 primarily accumulated in the mitochondria, likely due to insufficient ATP availability, leading to the buildup of HSP60 heptamers. Consequently, mitochondrial number and morphology were altered, protein levels in electron transport chain complexes were reduced, and oxidative phosphorylation was impaired. Additionally, reactive oxygen species accumulated, contributing to mitochondrial dysfunction in skeletal muscles. The upregulation of Pink-1/Parkin triggered enhanced autophagy, while increased Bax and poly (ADP-ribose) polymerase (PARP) cleavage mediated heightened apoptosis; both mechanisms aimed at eliminating damaged mitochondria. However, prolonged HSP60 accumulation overwhelmed these protective processes, ultimately leading to skeletal muscle dystrophy and premature death. Our findings demonstrated that excessive mitochondrial HSP60 initially boosts oxidative respiration; however, over time, it contributes to mitochondrial dysregulation and myopathy. This study provides novel insights into how excessive HSP60 affects mitochondrial oxidative respiration and glycolysis, with potential links to certain mitochondria-related diseases.

## Introduction

Mitochondrial heat shock proteins (HSPs) play a crucial role in chaperone systems that facilitate the import, folding, and removal of mitochondrial proteins, essential for maintaining mitochondrial homeostasis [1–3]. Heat shock proteins (HSP60), which interact with many mitochondrial peptides, are the central component of the chaperone system [3, 4]. This nucleus-encoded *HSP60* gene varies in expression levels across different health conditions [5–7]. When cells experience oxidative stress, HSP60 helps maintain cellular balance by preventing protein aggregation and assisting in the refolding of denatured proteins [3, 8, 9]. Its induction is recognized as a marker of the unfolded protein response within mitochondria, which triggers stress pathways that can lead to inflammation and cell death [7, 8, 10].

While complete HSP60 knockout in specific tissues or organs leads to diseases and death in animal models and humans [11–14], low HSP60 expression has been observed in various diseases, including sarcopenia [15], diabetes [16, 17], heart failure [18, 19], neurodegenerative diseases [20], and some types of cancers [21]. Conversely, elevated HSP60 levels are associated with inflammation and immune-related diseases, such as arthritis [22], atherosclerosis [23, 24], infections [25], and several cancers [26–28].

Strategies to increase cellular HSP60 levels have shown promising results in cell cultures and animal models [29–36]. However, the long-term effects of HSP60 induction in animals remain underexplored, as only a few studies have reported the use of HSP60 transgenic mice [29, 30, 34, 35]. Using the LoxP-Cre approach, we previously demonstrated that the overexpression of human HSP60 in mice led to neonatal death with underdeveloped hearts and septal defects [30]. In another study, we showed that heart-specific HSP60 induction in adult mice provided temporary protection during episodes of ischemia and regeneration, thereby preserving heart contractility [29]. These findings suggest that the outcomes of HSP60 induction may depend on both the dose and duration of expression. In this study, we used the LoxP-Cre approach to express varying levels of mouse HSP60 (mHSP60). mHSP60 was chosen to avoid potential issues arising from differences between the human and mouse forms of the protein.

## Results

### Excessive mHSP60 expression impairs growth and skeletal development

**Fig. 1:**
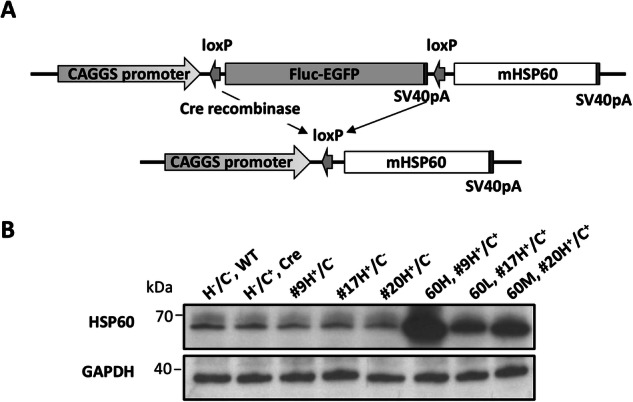
Generations of conditional mHSP60 transgenic mice. **A** Transgenic vectors control mHSP60 protein expression. Fluc-eGFP cDNA and SV40 polyA sequences are inserted between the two LoxP sites to prevent downstream transgene expression. The transgene comprises full-length mHSP60 cDNA with endogenous stop codons. The two LoxP sites are joined by Cre DNA recombinase, which enables mHSP60 protein expression. **B** Western blot analysis of skeletal muscles of 6-week-old transgenic mice generated from different founders. The lines include wild-type (H^−^/C^−^), CRE (H^−^/C^+^), and mHSP60 (H^+^/C^−^) lines #9, #17, and #20, and double transgenic lines (H^+^/C^+^) #9 (60H), #17 (60L), and #20 (60M).

We created a transgenic mouse model, G-Lox**-**mHSP60, which displayed enhanced green fluorescent protein (eGFP) driven by the cytomegalovirus immediate early enhancer and chicken β-actin promoter (CAGGS) (Fig. 1A). Three mouse lines, #9, #17, and #20, were studied, and all three lines were developed, weighed, and reproduced normally. mHSP60 expression was directly enhanced by CAGGS treatment through Cre-mediated recombination (Fig. 1A). The offspring of the three mHSP60 lines, 60H (#9 H^+^/C^+^), the high expression line; 60 L (#17 H^+^/C^+^), the low expression line; and 60M (#20 H^+^/C^+^), the medium expression line, expressed varying levels of mHSP60 in their skeletal muscles. The mHSP60 expression levels in 6-week-old mice are shown in Fig.1B.

**Fig. 2:**
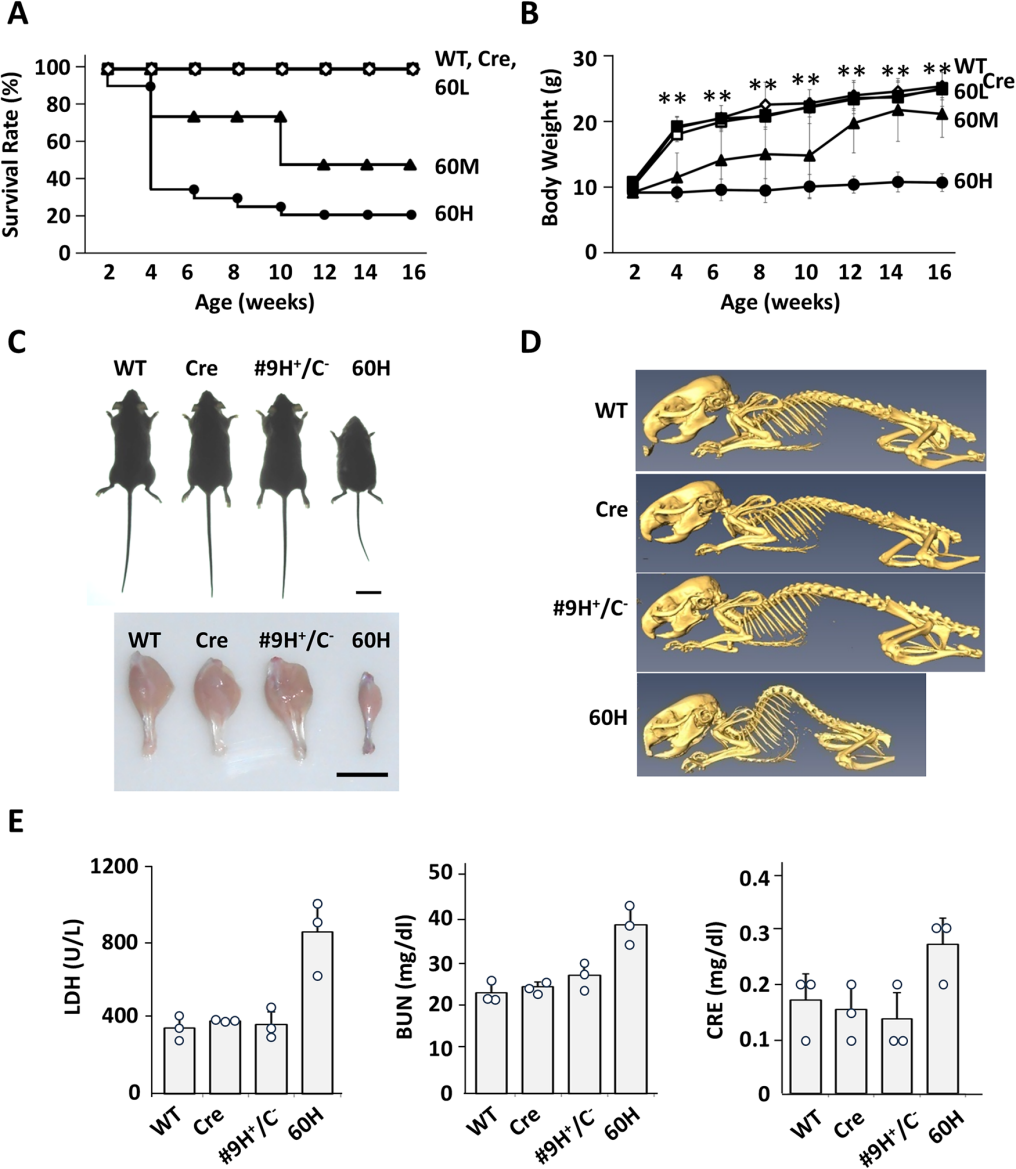
Characteristics of murine conditional mHSP60 mice. **A** Survival rates of conditional mHSP60 (60H) mice (female; WT, *n* = 12; Cre, *n* = 13; 60L, *n* = 12; 60H, *n* = 12. male; WT, *n* = 12; Cre, *n* = 11; 60L, *n* = 12; 60H, *n* = 12). **B** Body weights of conditional mHSP60 (60H) mice (female; WT, *n* = 12; Cre, *n* = 13; 60L, *n* = 12; 60H, *n* = 12). Values represent mean ± s.d. Analysis of variance (ANOVA): **p < 0.01. **C** Body sizes of mHSP60 transgenic and littermate control mice at 6 weeks old. The image shows whole-mount gastrocnemius muscles of 6-week-old mHSP60 (60H) and littermate control mice. The scale bars represent 1 cm. **D** 3D computed tomography image of mHSP60 transgenic mice and littermate control mice at 16 weeks old. **E** Biochemical blood analysis. Samples were collected from 6-week-old mHSP60 (60H) and littermate control mice. LDH lactate dehydrogenase, BUN blood urea nitrogen, CRE creatinine (*n* = 3). Values represent mean ± s.d.

The severity of the observed phenotypes was correlated with mHSP60 expression. The 60H line, which showed high mHSP60 transgene expression, had a mortality rate exceeding 75% at 10 weeks (Fig. 2A). 60H mice maintained normal weight during early development, but experienced significant weight loss after 3–4 weeks of age, coinciding with the onset of muscle pathology. This suggests that mHSP60 overexpression affects both mouse development and maturation and causes muscle degeneration. These mice weighed only 40–60% of the control mice as early as 4–6 weeks old and demonstrated severe muscle atrophy (Fig. 2B, C). Computed tomography revealed that the 60H mouse displayed skeletal abnormalities, whereas the Cre mouse and #9 H^+^/C^−^ mice without recombination were normal, similar to wild-type mice (Fig. 2D). Elevated levels of lactate dehydrogenase (LDH), blood urea nitrogen (BUN), and creatinine were observed in 60H mice, but not in other mice, indicating possible skeletal muscle damage and kidney failure in 60H mice (Fig. 2E).

### Excessive mHSP60 increases oxidative stress and skeletal myopathy

**Fig. 3:**
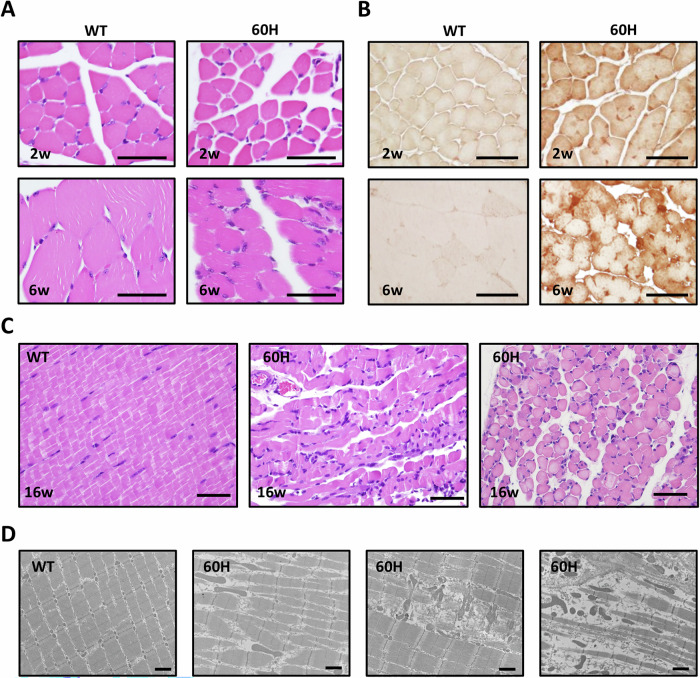
Transgenic expression of mHSP60 increased oxidative stress and skeletal myopathy. **A** Hematoxylin and eosin (H&E) staining of skeletal muscles from 2-week and 6-week-old mHSP60 (60H) mice and littermate controls reveals diffuse, moderately smaller myocytes and hypoplasia. Scale bars correspond to 20 μm. **B** This image depicts the detection of oxidative stress in the skeletal muscles of 2-week and 6-week-old mHSP60 transgenic mice (60H, right). Scale bars correspond to 20 μm. **C** H&E staining of skeletal muscles from 16-week-old mHSP60 (60H) mice and littermate controls reveals dystrophic changes and an increased number of nuclei. Scale bars correspond to 20 μm. **D** Transmission electron microscopy images of skeletal muscle sections from 16-week-old wild-type or mHSP60 transgenic mice (60H). Scale bars correspond to 1 μm.

Physical examination revealed that limb muscles of 60H mice were apparently dystrophic (Fig. 2B) compared to wild-type, Cre, or #9H^+^/C^−^ mice. Hematoxylin and eosin (H&E) staining revealed atrophic fibers in 60H mice as early as 2 weeks of age. At 6 weeks, 60H muscle fibers showed moderate-to-severe hypoplasia with diffuse changes (Fig. 3A). To determine whether mHSP60 overexpression in 60H cells resulted in mitochondrial damage or dysfunction, typically manifested as accumulation of reactive oxygen species (ROS), we performed ROS staining and transmission electron microscopy (TEM) for ultrastructural examination. Compared to wild-type control mice, both 2-week and 6-week-old 60H mouse fibers contained increased ROS, and the excess ROS was higher in 6-week-old fibers (Fig. 3B). The 6-week-old mice were used as a “late” time point for more advanced molecular and cellular changes, whereas 2-week-old mice were used as an early time point. We found that the mice needed to be euthanized at 6-8 weeks old. A few mice reached 16 weeks of age, and we were interested in why these mice survived, whereas others did not. We used H&E staining to visualize their anatomy and collected their muscles for pathological evaluation to assess ultra-late phenotypes or long-term compensatory outcomes. At 16 weeks, 60H myofibers exhibited moderate-to-severe hypoplasia with diffuse changes. Some fibers contained multiple internalized myonuclei (Fig. 3C). The TEM results revealed irregular shapes and disarray of microfibers and features of damaged mitochondria, such as structural disintegration and loss of an electron-dense matrix in the 60H muscle (Fig. 3D).

### Excessive mHSP60 *leads to* incomplete assembly of HSP60 chaperones

**Fig. 4:**
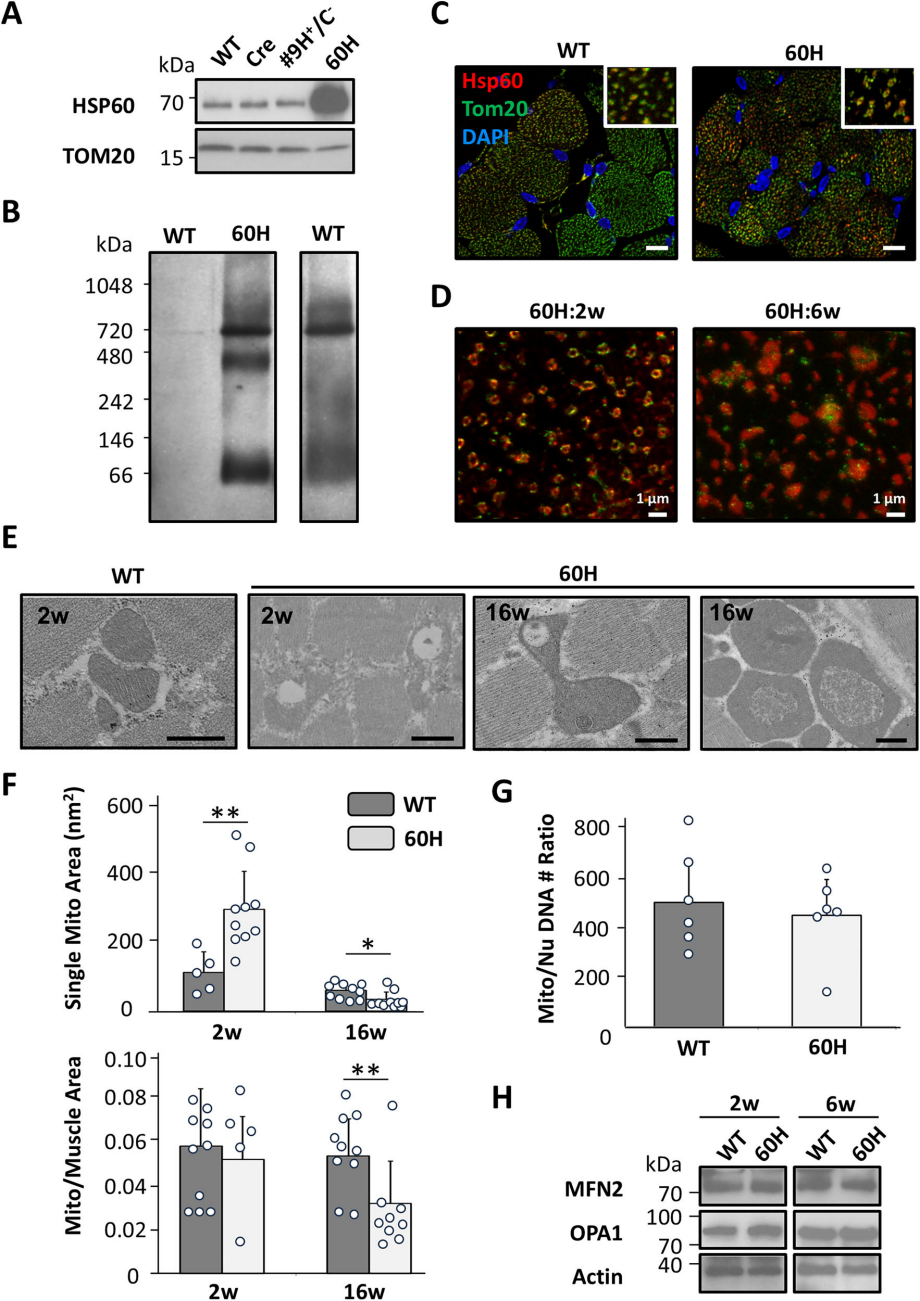
Expression, folding, and distribution of mHSP60 in the mitochondrion. **A** Western blot analysis of skeletal muscle mitochondrial fractions. **B** Analysis of skeletal muscle mitochondrial fraction using BN-PAGE and western blotting. The figure on the right shows a longer exposure of the wild-type (WT) muscle fraction. BN-PAGE, blue native polyacrylamide gel electrophoresis. **C** Immunofluorescence staining reveals increased mHSP60 expression and its localization in 2-week-old muscle cross-sections. HSP60 is labeled red using Alexa Fluor 568-conjugated IgG, and TOM20, a mitochondrial pre-protein translocase of the outer membrane, was labeled green using FITC-conjugated IgG. The cells were counterstained with 4′,6-diamidino-2-phenylindole (DAPI). Bars represent 10 μm. **D** High-resolution microscopic images (STED; Leica). Scale bars correspond to 1 μm. **E** Transmission electron microscopy (TEM) of skeletal muscle sections from 2-week-old (WT) and 2- and 16-week-old mHSP60 (60H) mice. Scale bars correspond to 500 nm. **F** Quantification of mitochondrial size. The mitochondrial area was measured in at least 10 representative EM fields. Single mitochondrial area was quantified at 2 weeks (WT, *n* = 5; 60H, *n* = 10) and 16 weeks (WT, *n* = 10; 60H, *n* = 10). Mito/muscle area was quantified at 2 weeks (WT, *n* = 10; 60H, *n* = 5) and 16 weeks (WT, *n* = 10; 60H, *n* = 9). Values represent mean ± s.d. Analysis of variance (ANOVA): *p < 0.05; **p < 0.01. G. Relative mtDNA copy number compared to nuclear DNA in 2-week-old mHSP60 (60H) and WT mice (WT, *n* = 6; 60H, *n* = 6). Values represent mean ± s.d. **H** Western blot analysis of 2- and 6-week-old mHSP60 skeletal muscle using MFN2, OPA1, and actin antibodies.

mHSP60 protein levels were significantly increased in the mitochondrial fraction of 60H mice muscles compared to those in wild-type, Cre, and #9 H^+^/C^−^ mice (Fig. 4A). After being imported into the mitochondrial matrix, HSP60 assembles into a single heptameric ring structure from seven HSP60 monomers and interacts with HSP10 in the head region [1, 37]. The two HSP60 heptamers were further assembled into tetradecamers as fully functional chaperones. To investigate the assembly of transgenically expressed mHSP60 into tetradecamers in mouse tissue, we performed blue native polyacrylamide gel electrophoresis (BN-PAGE)/western blot analysis of the muscle mitochondrial fractions. The results showed that transgenic mHSP60 proteins formed tetradecamers in large quantities; however, in addition to tetradecamers, there was an excess of mHSP60 monomers and heptamers (Fig. 4B). In contrast, the wild-type mouse muscle mitochondrial fractions contained only mHSP60 tetradecamers and monomers, and intermediate mHSP60 heptamers were not visible by western blotting. In 60H mouse mitochondria, the large quantity of newly synthesized/imported mHSP60 may have overwhelmed the normal mHSP60 assembly capacity. Thus, the amount of mHSP60 tetradecamers that could be formed was likely limited by the available number of assembling cofactors, such as HSP10 and ATP, leading to the accumulation of the intermediate mHSP60 heptamers. Mitochondrial dysfunction related to impaired oxidative phosphorylation and ATP production further aggravates the excessive accumulation of mHSP60 heptamers.

### Excessive mHSP60 altered mitochondrial morphology

Immunofluorescence staining was used to investigate the locations of transgenic mHSP60. The results revealed that mHSP60 was primarily localized in the mitochondria, co-localizing with the mitochondrial marker TOM20 (Fig. 4C, D). The mHSP60 in 2-week-old 60H mice displayed a cylindrical shape, with TOM20 intercalated on the outer surface of the ring. In 6-week-old 60H mouse muscle, mHSP60 appeared as solid irregular shapes, increased in size, and lacked the cylindrical and ring structures observed in 2-week-old mice, and the presence of TOM20 reduced (Fig. 4D). As shown in Fig. 4D, relative quantification of HSP60 fluorescence revealed a significant increase from 2 weeks (1.00 ± 0.28) to 6 weeks (2.93 ± 1.55; *p* < 0.01), indicating an age-dependent upregulation of mitochondrial stress response. The presence of mitochondrial swelling in the 60H mouse muscle was shown by TEM (Fig. 4E). Excess mHSP60 leads to mitochondrial swelling, loss of contrast, vacuolation, and mitochondrial fusion. In 2-week-old mHSP60 (60H) mice, the average area of a single mitochondrion was larger than that in wild-type mice. However, after normalization to muscle area, the mitochondrial area per muscle area region was not different from that in wild-type mice (Fig. 4F), indicating that the appearance of larger mitochondria was, at least in part, due to fusion. In 16-week-old mHSP60 (60H) mice, both the single mitochondrial area and normalized mitochondrial area per muscle area were smaller than those in the same mice at a younger age, and smaller than those in wild-type mice. Furthermore, we measured mitochondrial DNA (mtDNA) copy number in 60H mice and found that the mtDNA copy number relative to nuclear DNA was similar to that in 2-week-old wild-type mice (Fig. 4G). We checked the expression of proteins related to mitochondrial fusion and found a slight increase in Mfn2 and OPA1 expression in 2- and 6-week-old 60H mouse muscles compared to wild-type mouse muscles (Fig. 4H).

### Excessive mHSP60 leads to reduced electron transport chain (ETC) complex activity

**Fig. 5:**
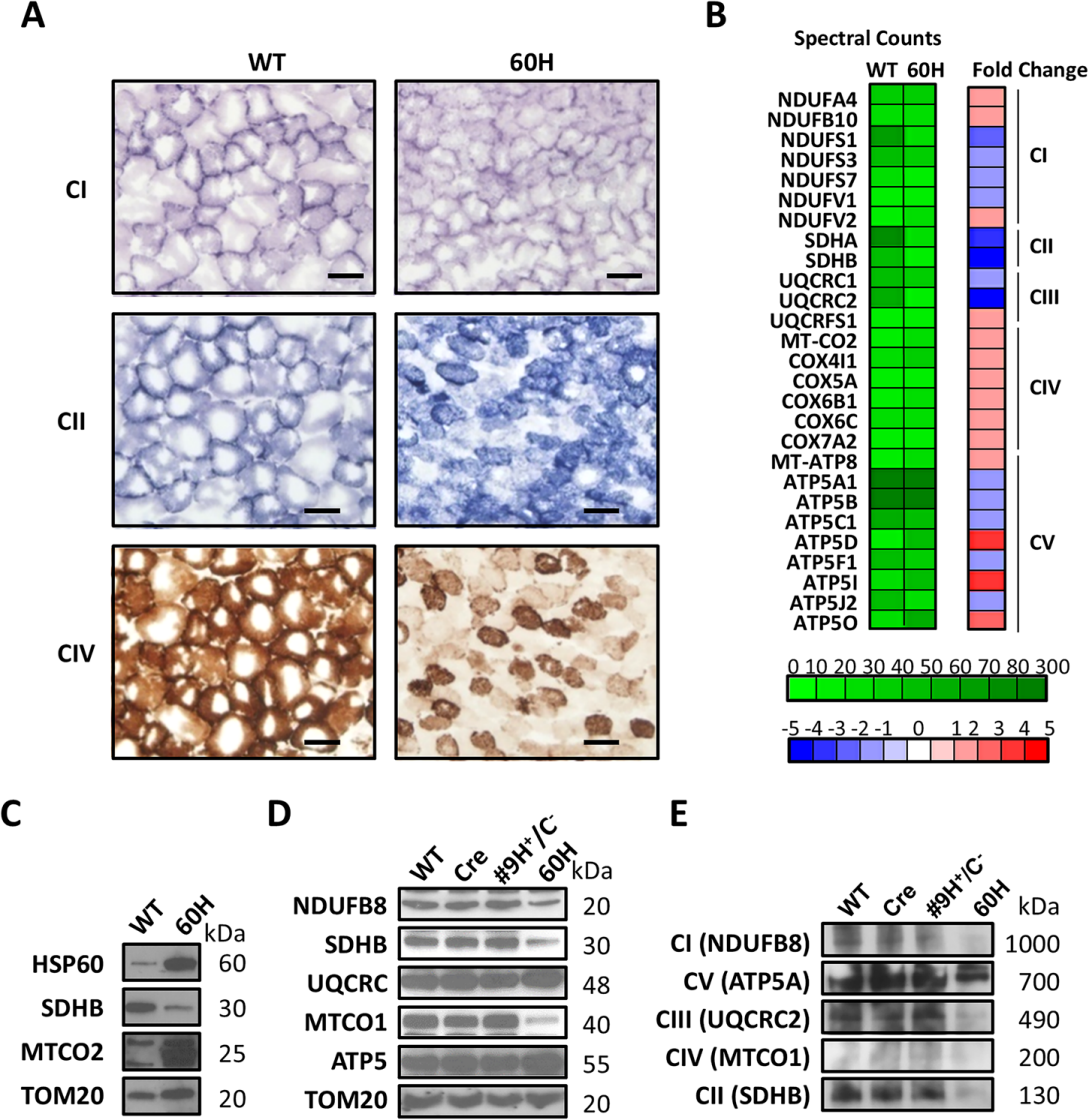
Excessive mHSP60 alters protein levels in mitochondrial respiratory enzymes and metabolism. **A** This panel displays the activities of NADH (complex I, blue), SDH (complex II, blue), and COX (complex IV, brown) in tissue sections of freshly isolated 2-week-old transgenic mouse skeletal muscles. The scale bars correspond to 20 μm. **B** Mitochondrial proteins identified and quantified using one-dimensional mass spectrometry and spectral counts, respectively. The heat map shows fold changes compared to the wild-type tissue. Significant relative spectral count-fold-change ratios (Rsc) were calculated using a modified formula from Beissbarth et al.‘s 2004 study [58]. **C** Verification of mitochondrial proteins with SDHB and MTCO2 antibodies and specific mitochondrial markers. **D** This panel illustrates the levels of oxidative phosphorylation (OXPHOS) complex proteins in the mitochondria of control and mHSP60 transgenic mice. TOM20 was used as the reference for protein loading. **E** This panel presents a native polyacrylamide gel electrophoresis (PAGE)/western blot with an anti-OXPHOS complex antibody. The indicated molecular weights are in kilodaltons (kDa).

Analysis of individual complex oxidative phosphorylation activities revealed that mHSP60 overexpression decreased the enzymatic activities of key mitochondrial respiratory enzymes (complexes I, II, and IV) in skeletal muscle bundles compared to that in the wild-type muscle (Fig. 5A). The decrease in complex I and IV activities was more significant and uniform across the muscle section than that in complex II, where the decrease appeared to occur in certain muscle fibers and remained unchanged in other fibers. The expression of complex components was analyzed using nano-liquid chromatography-tandem mass spectrometry (LC–MS/MS)-based peptide identification, and the mitochondrial fractions from the skeletal muscles of 2-week-old 60H and wild-type mice were compared. Significant changes in 33 mitochondrial respiratory enzymes were identified in 60H mouse muscles at 2 weeks of age (Fig. 5B). The peptides with the most significant decreases were nicotinamide adenine dinucleotide (NADH) ubiquinone oxidoreductase subunit (NDUF)S1 of complex I, succinate dehydrogenase (SDH)A and SDHB of complex II, and ubiquinol-cytochrome C reductase core protein (UQCRC)2 of complex III. In contrast, ATP5D, ATP5I, and ATP5O peptides of complex V were significantly increased in 60H mitochondria. Among these proteins, the mitochondria-encoded cytochrome C oxidase (MTCO)2 of complex IV and ATP8 of complex V are encoded by the mitochondrial genome. Western blot analysis confirmed the differential expression of SDHB and MTCO2, which were initially identified by proteomic analysis (Fig. 5C). The validation results strongly correlated with the protein profiles obtained from our mass spectrometry data. This implies that mitochondrial dysfunction caused by mHSP60 overexpression could extend beyond nucleus-encoded and imported proteins, potentially affecting mitochondrial transcription and translation. We used sodium dodecyl-sulfate polyacrylamide gel electrophoresis (SDS–PAGE) and western blotting to confirm the LC–MS/MS results. We found that NDUFB8 (complex I), SDHB (complex II), and MTCO1 (complex III) expressions were significantly reduced, whereas those of UQCRC (complex III) and ATP5 (complex V) remained unchanged or slightly increased (Fig. 5D). Furthermore, we separated the oxidative respiration complex (complexes I to V) by BN-PAGE according to molecular weight, followed by the identification of their sizes using western blot analysis. The abnormal assembly of complexes results in a component of the complex with a different molecular weight. Compared to all other control mice, the mitochondrial fraction of 60H mouse muscle showed a significant reduction in NDUFB8 (complex I), SDHB (complex II), UQCRC2 (complex III), MTCO1 (complex IV), and ATP5A (complex V); however, the locations of the complexes appeared to be the same as in control mice (Fig. 5E). The levels decreased to only 0.467, 0.520, 0.546, 0.742, and 0.960 of their normal values, respectively. Taken all expression analyses together, in 60H mice, complexes I–IV appeared to be most affected by mHSP60 overexpression, whereas complex V was less affected.

### Excessive mHSP60 induces apoptosis and autophagy in skeletal muscles

**Fig. 6:**
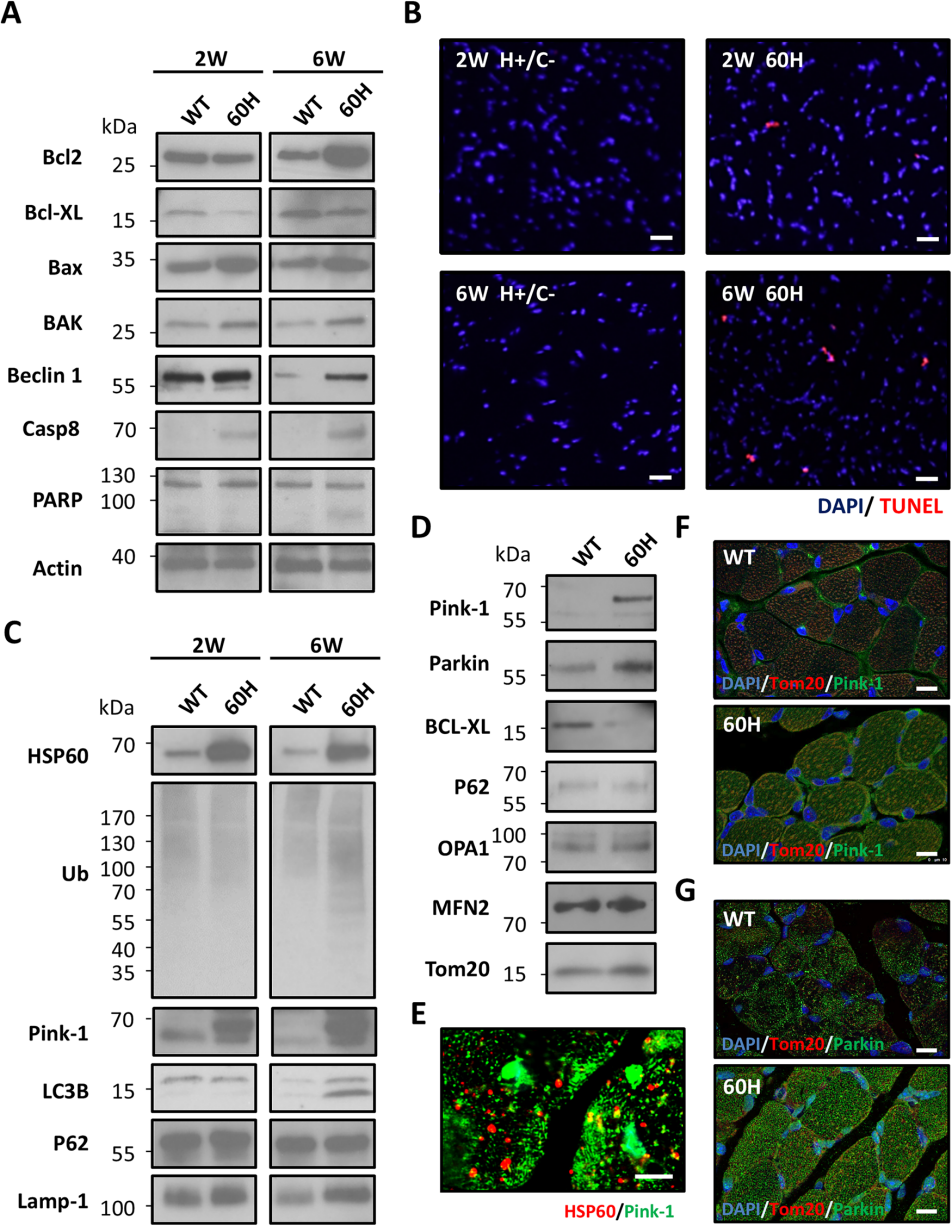
Apoptosis and autophagy in skeletal muscles of mHSP60 transgenic mice. **A** Immunoblot analysis of Bcl2, Bcl-XL, Bax, Beclin 1, Casp8, PARP, and Actin expression in the skeletal muscles of WT and mHSP60 (60H) mice at 2 or 6 weeks of age. **B** Detection of nuclear DNA fragmentation with TUNEL reaction in skeletal muscles of WT and mHSP60 (60H) mice at 2 or 6 weeks old. Fluorescein-12-dUTP (TdT) and 4′,6-diamidino-2-phenylindole (DAPI) were used for TUNEL and nuclear staining, respectively. Scale bars correspond to 20 μm. **C** Immunoblot analysis of HSP60, ubiquitin (Ub), Pink-1, LC3B, SQSTM1/P62 (P62), and LAMP-1 expressions in the skeletal muscles of WT and mHSP60 (60H) mice at 2 or 6 weeks of age. **D** Immunoblot analysis of Pink-1, Parkin, Bcl-XL, P62, OPA1, MFN2, and TOM20 expressions in the mitochondria of WT and mHSP60 (60H) mice at 2 weeks of age. **E** Confocal images showing anti-Parkin, anti-Pink-1 (labeled with green Alexa Fluor 488 secondary antibody), and anti-HSP60 (labeled with red Alexa Fluor 594 secondary antibody) in the skeletal muscles of 2-week-old 60H mice. Scale bars represent 10 μm. **F** Confocal images showing anti-Pink-1 (labeled with green Alexa Fluor 488 secondary antibody) and anti-TOM20 (labeled with red Alexa Fluor 594 secondary antibody) in the skeletal muscles of 2-week-old 60H mice. Nuclei were counterstained with DAPI (blue). Scale bars correspond to 10 μm. **G** Confocal images showing anti-Parkin (labeled with green Alexa Fluor 488 secondary antibody) and anti-TOM20 (labeled with red Alexa Fluor 594 secondary antibody) in the skeletal muscles of 2-week-old 60H mice. Nuclei were counterstained with DAPI (blue). Scale bars correspond to 10 μm.

Mitochondrial impairment and dysfunction often lead to autophagy and/or apoptosis, which limit damage at the organelle and cellular levels. We studied the expression of a few apoptosis-related protein markers and found that Bcl-2, Bax, and BAK were significantly increased in 60H mouse muscle at 2 weeks of age and further increased at 6 weeks of age. Interestingly, the expression pattern of Bcl-XL, an anti-apoptotic protein, was reversed and was significantly lower in 60H mice than in wild-type mice (Fig. 6A). Beclin 1, a tumor suppressor that interacts with Bcl-2, Bax, BAK, Casp8, and Bcl-XL, and triggers apoptosis and induces autophagy, was significantly increased in 60H muscle compared to that in age-matched wild-type mice. Furthermore, cleaved-PARP fragments were clearly observed in the muscles of 6-week-old 60H mice, but not in 2-week-old mouse (Fig. 6A). These findings agreed with the DNA fragmentation results, showing increased TUNEL-positive nuclei in 60H mouse muscles, which were more pronounced at 6 weeks of age than at 2 weeks of age (Fig. 6B). The levels of SQSTM1/p62 and LAMP1, and the presence of LC3B-II and Pink-1 (PTEN-induced putative kinase 1) were studied for the production of autophagosomes and activation of the lysosomal axis. Both p62 and LAMP1 increased in 2-week-old 60H mice muscles, whereas LC3B-II became evident at 6 weeks of age (Fig. 6C). Furthermore, an increase in ubiquitinated proteins in the 6-week-old 60H muscle indicates that proteasomal stress was also likely present in this muscle. This suggests that excess mHSP60 activates the ubiquitin-proteasome system and employs the proteasome system for its degradation. Excess mHSP60 may have depleted the normal proteasomal capacity and caused the accumulation of ubiquitinated proteins in 6-week-old 60H muscles. Muscle cell death and inflammation in the dystrophic muscles of 60H mice at a later stage likely involve unfolded protein responses.

Furthermore, we studied whether mitophagy occurred in 2-week-old 60H muscles and found that Pink-1 and Parkin significantly increased, whereas Bcl-XL was significantly decreased in the muscle mitochondrial fraction compared to wild-type mice (Fig. 6D). Other markers, such as p62, OPA1, and MFN2, did not change significantly at this time point. This result suggests that excess mHSP60 proteins cause mitochondrial damage by activating Pink-1, which recruits Parkin to damaged mitochondria for degradation via mitophagy. Pink-1/Parkin-dependent mitophagy was further facilitated by a reduction in Bcl-XL levels, which was also observed in the 60H mice. Bcl-XL, BAK, Bax, and Beclin-1 are known direct binding partners to HSP60; thus, autophagy and apoptosis, involving or resulting from changes in these molecules, may be directly or indirectly related to mHSP60 overexpression. In the skeletal muscle of 2-week-old mice exposed to 60H, Pink-1 co-localized with mHSP60 (Fig. 6E). Additionally, the co-localization of Pink-1 and Parkin with mitochondria increased significantly, supporting the activation of mitophagy (Fig. 6F, G). ImageJ software was used to quantify colocalization. Compared to WT mice, 60H mice showed a 4.16-fold increase in Pink-1 colocalization with mitochondria and a 1.69-fold increase in Parkin colocalization. These significant increases in both indicators suggest that Pink-1/Parkin enhances mitochondrial targeting.

### Excessive mHSP60 leads to an increase in respiration and glycolytic capacity in 2-week-old skeletal myoblasts

**Fig. 7:**
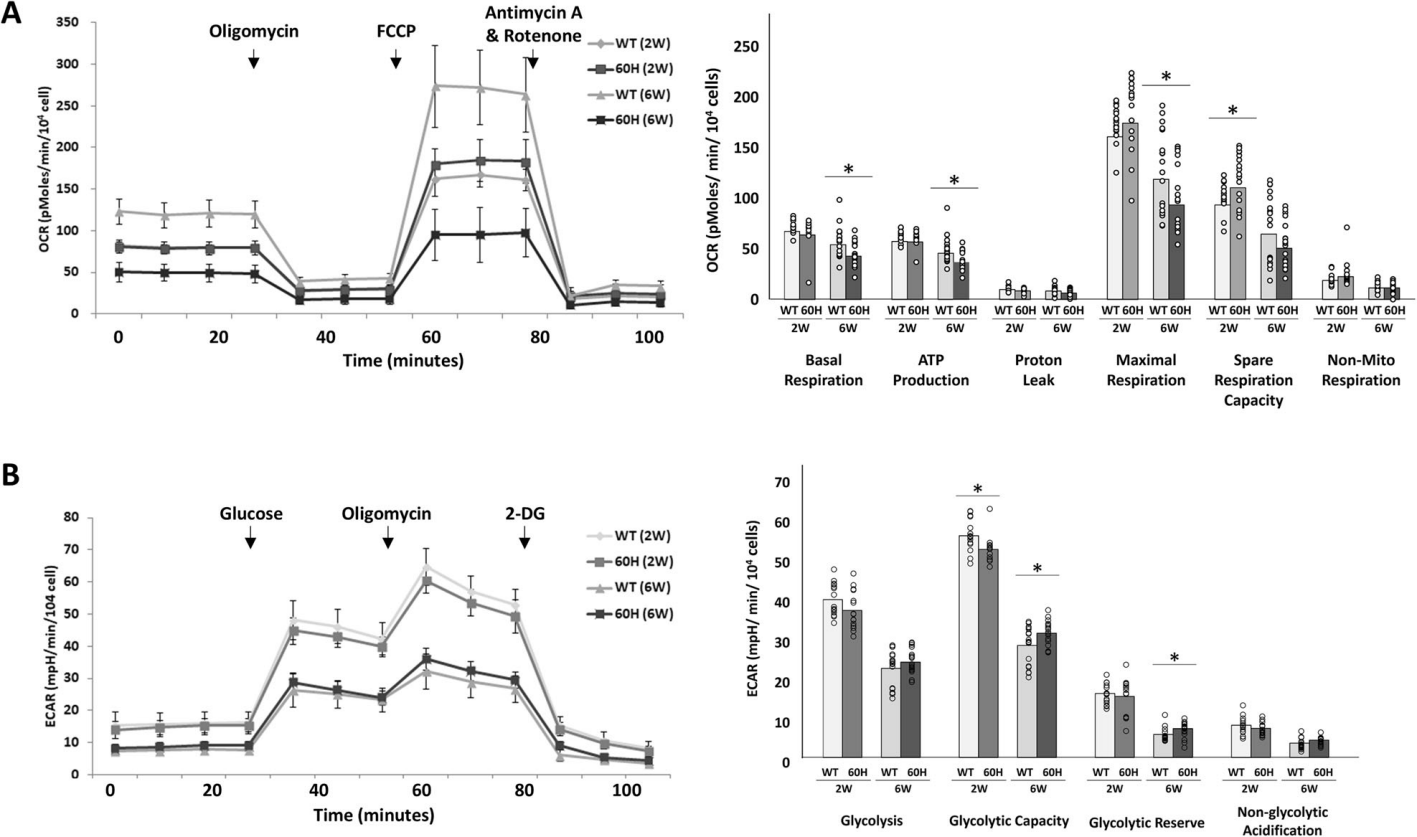
Oxygen consumption and glycolytic capacity of excessive mHSP60 myoblasts. **A** Oxygen consumption rates (OCR) of wild-type (WT) and mHSP60 (60H) mouse skeletal myoblasts (passage 2) isolated from 2- or 6-week-old mice under basal conditions and mitochondrial stress. The stress was induced by sequential addition of oligomycin (0.5 µM), carbonyl cyanide-p-trifluoromethoxyphenylhydrazone (FCCP, 1 μM), and Antimycin A/Rotenone (0.5 mM) at 25 min intervals. The OCR values for basal respiration, ATP production, maximum respiration, spare respiratory capacity, proton leakage, and non-mitochondrial respiration were calculated according to the manufacturer’s protocol (*n* = 18). The summarized results are displayed below. Values represent mean ± s.d. Analysis of variance (ANOVA): *p < 0.05. **B** Extracellular acidification rate (ECAR) of myoblasts from 2- or 6-week-old mice under different conditions: basal conditions, saturated glucose, maximized glycolytic capacity, glycolytic activity reserve, and non-glycolytic acidification. Myoblasts were obtained from WT and mHSP60 (60H) mice. During the glycolysis stress test, the myoblasts were cultured in a glucose-free medium and treated with sequential addition of glucose (10 mM), oligomycin (1 µM), and 2-deoxy-D-glucose (50 mM) (*n* = 18). Values represent mean ± s.d. ANOVA: *p < 0.05.

To explore the effect of mHSP60 overexpression on mitochondrial function, including respiration and glycolysis, we performed Seahorse extracellular flux analysis and measured the oxygen consumption rate (OCR) and extracellular acidification rate (ECAR). Although measuring the OCR and ECAR of fresh mouse muscle sections has been demonstrated elsewhere, consistent results and stable experimental conditions using fresh muscle sections were not achieved in this study. Thus, we alternatively measured the OCR and ECAR of primary isolated skeletal myoblasts from 60H and wild-type mice. Skeletal myoblasts isolated from different mouse stages displayed age-dependent characteristics, including a reduction in OCR and glycolysis in myoblasts isolated from 6-week-old mice compared with myoblasts isolated from 2-week-old mice. In 6-week-old myoblasts, mHSP60 overexpression further reduced most parameters related to basal respiration, ATP production, and maximal and spare respiration capacity; however, mHSP60 increased glycolytic capacity (Fig. 7A, B). Interestingly, in 2-week-old myoblasts overexpressing mHSP60, the respiration capacity was not as reduced as in 6-week-old myoblasts; instead, an increase in the maximal and spare respiration capacity and a decrease in glycolytic capacity and spare glycolytic capacity were observed. These data suggest that in the early stages, mHSP60 overexpression in myoblasts enhances the respiratory capacity of mitochondria at the cost of glycolytic capacity. The mitochondria become impaired over time by mHSP60 overexpression, leading to reduced respiratory capacity and a compensatory increase in glycolytic activity.

## Discussion

This study showed that increased mHSP60 expression impairs postnatal development and promotes skeletal muscle dystrophy. The severity of the phenotype depended on the mHSP60 transgenic dose, with over 70% mortality observed at 10 weeks of age in 60H mice. Significant increases in blood LDH and creatinine levels indicated muscle damage (Fig. 2D). In 60H mice, mitochondrial dysfunction and a reduction in ETC complex activity were observed in the skeletal muscle, leading to the accumulation of lactic acid and increased muscle acidity, implying anaerobic ATP production. HSP60 also plays a role in autoimmune diseases and T cell activation [38]. Following myocyte death at a late stage, HSP60 is believed to be released from damaged cells and triggers immune cell receptors such as TLR4 and CD14 [39]. These receptors activate pro-inflammatory pathways and function in damage-associated molecular patterns [40], causing further inflammation and tissue damage. The changes in mitochondrial oxidative respiration and glycolysis pathways by HSP60 could be a mechanism underlying certain mitochondria-related diseases.

**Fig. 8:**
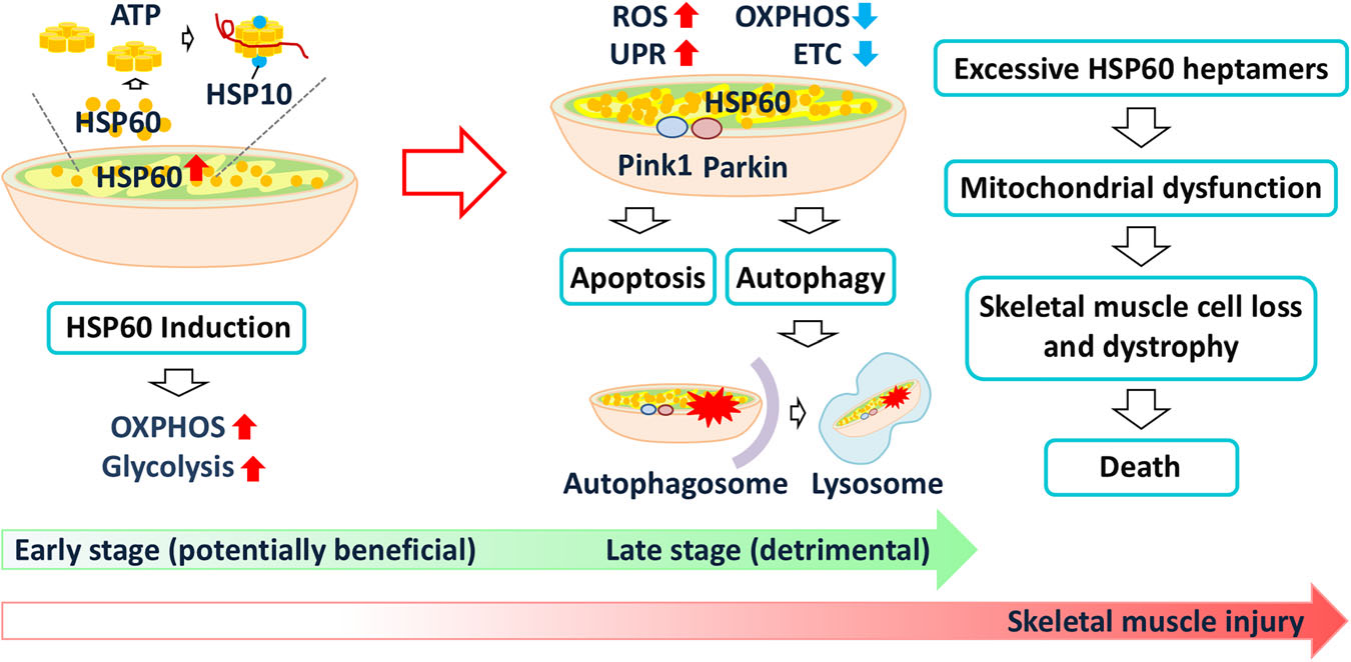
Mechanism of excessive mHSP60 causing mitochondrial damage, leading to skeletal myopathy and premature death.

The immediate and long-term effects of mHSP60 overexpression in mice are shown in Fig. 8. Initially, mHSP60 accumulated primarily in the mitochondria, enhancing chaperone assembly and oxidative respiratory capacity. However, increased OXPHOS activity leads to higher ROS levels, eventually exceeding the capacity of antioxidants (such as glutathione) to eliminate ROS effectively [41]. Elevated ROS and disturbed mitochondrial proteostasis due to excessive mHSP60 accumulation caused mitochondrial damage with reduced respiration capacity and triggered Pink-1-Parkin-mediated mitophagy. Apoptosis triggered by damaged mitochondria or excess mHSP60 further contributes to myocyte loss, causing muscular dystrophy and premature death (Fig. 8).

HSP60 requires the help of co-chaperones HSP10 and ATP for proper folding into heptamers and tetradecamers [2, 37, 42, 43]. mHSP60 overexpression increased HSP60 heptamers and monomers (Fig. 4B), which is a sign of a disrupted equilibrium among different forms of mHSP60 proteins. Disruption of the equilibrium between various forms of HSP60 is thought to reduce the efficiency of protein import and refolding machinery [44, 45], potentially leading to reduced OXPHOS and ATP production and impaired mitochondrial homeostasis (Figs. 5, 7A). Reduced ATP production in 60H myocytes, which demand extra ATP, further exacerbates mitochondrial dysfunction.

Many degenerative muscular diseases, including type II diabetes and aging-induced sarcopenia, share a common pathology in skeletal muscle, including reduced cell mass and cell numbers, loss of mitochondrial numbers, and reduction of HSP60 [46, 47]. Increased HSP60 expression has been proposed as a therapeutic strategy for aging-related diseases [48]. Furthermore, physical exercise (e.g., resistance training), which is known to greatly improve frailty and reverse the decline in muscle mass/strength in older adults, has been shown to induce HSP60, increase mitochondrial respiration and its efficiency, and enhance mitochondrial turnover and biogenesis [49].

However, the protective roles of HSP60 in vivo have only been demonstrated in surprisingly few reports. Transgenic mice that express an HSP60 fragment in bone marrow and thymus by MHC II-E*α* (HIIE*α*) promoter reduced susceptibility to autoimmune diabetes [35]. Transgenic mice overexpressing full-length HSP60 by mitochondrial phosphoglycerate kinase 1 promoter slowed the progression of osteoarthritis [50]. Transgenic mice expressing cytosolic HSP60 by omitting the mitochondrial recognition sequence showed resistance to liver stress and enhanced cell survival [33]. Previously, we showed that overexpressing human HSP60 leads to embryonic heart degeneration and apoptosis with massive hemorrhage, atrial septal defects, and neonatal death [30]. Although in this study, 60H mice developed muscle dystrophy at 6 weeks, and skeletal myocytes isolated from 6-week-old mice exhibited reduced respiration capacities, we found 60H mice to be free of pathological findings at 2 weeks old and observed increased respiration capacity in skeletal myoblasts. Our data suggest that targeting mHSP60 by titrating its increased expression remains a viable approach for degenerative muscle diseases.

This study had several limitations. First, although we did not include a control strain expressing a non-functional mitochondrial protein (e.g., mitoGFP), we attempted to address this by grading HSP60 expression at 60L, 60M, and 60H. Notably, only 60H mice (mice with the highest HSP60 levels) showed significant mitochondrial dysfunction and muscle pathology, whereas 60L and 60M mice did not show obvious phenotypes despite elevated HSP60 expression. These observations suggest that the pathological findings in 60H mice are likely caused by threshold-dependent effects specific to HSP60 accumulation, although general mitochondrial protein overexpression cannot be completely ruled out. Second, although MFN2 and OPA1 levels remained unchanged, we did not assess other key regulators of mitochondrial dynamics (e.g., DRP1, FIS1, and MFF). Although it is unlikely that these HSP60-specifically regulated regulators showed significant changes in expression and resulted in subsequent mitochondrial dysfunction, changes in mitochondrial dynamics associated with some of these regulators may have occurred during the transition from enhanced oxidative phosphorylation to hyporespiration and mitophagy, which led to the morphological and functional changes observed in 60H mouse muscles. Third, the interpretation of altered complex I and II activities relied on indirect measures. Direct enzymatic assays and high-resolution respirometry would provide stronger functional characterization. Finally, while the combined results of DNA fragmentation shown by positive TUNEL staining and cleaved PARP fragments serve as a strong indication of apoptotic event in 60H muscles, more comprehensive analyses, such as the detection of the phosphorylation status of BCL-2 family members, can confirm and provide mechanistic insight into the initiation of apoptosis. Future studies should incorporate conditional models, broader tissue profiling, and targeted molecular interventions to better understand mHSP60-specific contributions to mitochondrial pathology.

In conclusion, our findings demonstrated that excessive mitochondrial HSP60 initially boosts oxidative respiration; however, over time, it contributes to mitochondrial dysregulation and myopathy. This study provides novel insights into how excessive HSP60 affects mitochondrial oxidative respiration and glycolysis, with potential links to certain mitochondria-related diseases.

## Materials and methods

### Genetically engineered mice

LucG-Lox-mHSP60 mice were generated using a transgenic vector (Fig. 1A). This vector contains the CAGGS and two LoxP-flanked firefly luciferase cDNA fragments. We inserted these fragments using primer pairs (5′-TTGACCGGTCACCATGGAAGACGCCA AA-3′/5′-GCGACCGGTTTCAATTTGGACTTTCC-3′) from the pGL2 vector (Promega, Madison, WI). The vector also contained an eGFP-fused protein-coding sequence and SV40 PolyA. We inserted the mHSP60 mRNA (NM 010477.4, 148–1898 bp) into this using corresponding primer pairs (5′-GGGAAGCTTCCCCGCAGAAATGCTTC-3′/5′-CCAAGCTTGCAAAGCACTACTCTAGG-3′). Finally, the PCR fragment was cloned into the HindIII sites of the pCALELD vector [51, 52], resulting in the CAGGS-LoxP-FlucG-LoxP-mHSP60 vector.

All animal experiments were conducted according to the accepted standards of animal care. The mice were bred and maintained in pathogen-free facilities. To generate transgenic mice, we injected the CAGGS-LoxP-FlucG-LoxP-mHSP60 fragment into C57BL/6 blastocysts. These blastocysts were then transferred to surrogate mothers. The offspring were backcrossed with C57BL/6 mice to ensure germline transmission and generate CAGGS-LoxP-FlucG-LoxP-mHSP60 mice. We have previously described the process for generating Cre transgenic mice [53]. Transgenic mice were genotyped by tail biopsy using an Allele-In-One Mouse Tail Direct PCR System kit (Allele Biotech, San Diego, CA, USA). We used specific PCR primer pairs for genotyping: (LucG-Lox-mHSP60 allele: 5′-CATGGTCCTGCTGGAGTTCGTG-3′/5′-GAACCCTTAATATAACTTCGTATAAGTA TGC-3′; CRE allele: 5′-CATGGTCCTGCTGGAGTTCGTG-3′/5′-CCTGGTCGAAATCAG TGCGTTC-3′; and Recombinant allele: 5′-CTGCTAACCATGTTCATGCC-3′/5′-GGACTG TGGGTAGTCGAAGCATTTC-3′).

### Micro-computed tomography equipment

Exposures were conducted using a micro-X-ray computed tomography (micro-CT) unit (FLEX Triumph, GAMMA MEDICA-IDEAS, Northridge, CA), with a 60 kVp X-ray source. The unit allows high-resolution reconstructions down to 15 microns (X-O CT, GAMMA MEDICA-IDEAS). X-ray datasets are saved on the system’s hard drive and can be reconstructed either concurrently with the scan or afterwards. All micro-CT images were captured using a charge-coupled device with a field width of 2368 pixels and a length of 2240 pixels. The images were reconstructed using Shepp−Logan filters and real-time reconstruction.

### Blood chemistry assay

Blood samples were collected under anesthesia using capillary tubes and stored in heparinized tubes. These samples were analyzed using an automated clinical method to measure the levels of LDH, BUN, and creatinine using biochemical detection reagents.

### Histological and immunohistological analyses

The skeletal muscles of the mice were perfused with phosphate-buffered saline (PBS) and fixed with 10% formalin buffer. The paraffin-embedded muscle was then cut into serial 5 µm sections. H&E staining was conducted to evaluate the morphology and cellular dimensions.

An OxyIHC oxidative stress detection kit (S7450; Millipore, Burlington, MA, USA) was used to analyze skeletal muscle oxidation, following the manufacturer’s instructions. Formalin-fixed paraffin-embedded tissue sections were deparaffinized in xylene and rehydrated using a graded ethanol series. Antigen retrieval was performed by adding 250 µL of 1× Antigen Retrieval Buffer to each slide, followed by incubation in a preheated steamer for 20 min. The slides were then cooled to room temperature (RT) for 15 min. Tissue sections were washed in 250 µL of 1× Wash Buffer for 5 min, repeated twice. For oxidative modification detection, 250 µL of 2,4-dinitrophenylhydrazine solution was applied to each slide. Control sections received 250 µL of derivatization control solution. All the slides were incubated in a humidified chamber at RT in the dark for 30 min. After incubation, the slides were washed three times (5 min each) with 1× wash buffer at RT. Non-specific binding was blocked using 250 µL of 1× Blocking Buffer for 30 min in a humidified chamber. Subsequently, sections were incubated with 250 µL of freshly diluted primary antibody, covered with plastic coverslips, and maintained in a humidified chamber for 1–2 h at RT or overnight at 4°C. Following primary incubation, the slides were washed thrice with 1× Wash Buffer (5 min each). Tissue sections were then incubated with 250 µL of biotinylated secondary antibody for 30 min at RT. After another three washes in 1× Wash Buffer, endogenous peroxidase activity was quenched by applying 250 µL of 3% H₂O₂ for 10 min at RT, followed by an additional three wash steps. Detection was carried out using 250 µL of streptavidin-conjugated horseradish peroxidase, incubated for 30 min at RT in a humidified chamber. After washing (3 × 5 min, RT), 250 µL of freshly prepared 3,3′-diaminobenzidine substrate solution (components A and B) was applied for 2–5 min at RT until optimal staining intensity was achieved. The slides were dehydrated in 100% ethanol (2 × 5 min), cleared in xylene (3 × 5 min), and mounted using a permanent mounting medium. Sections were air-dried overnight before microscopic examination under bright-field illumination.

The TUNEL assay was used to detect nuclear DNA fragments as an indicator of apoptosis, using Fluorescein-12-dUTP as a substrate (DeadEnd Fluorometric TUNEL System, Promega).

For immunostaining, the tissue sections were deparaffinized with xylene, rehydrated using graded alcohol solutions, and rinsed with diH_2_O and 1× Tris-buffered saline using standard protocols. Antigen retrieval was performed in a microwave oven for 15 min with Tris–ethylenediaminetetraacetic acid at pH 9.0. The fixed sections were blocked in a solution (5% normal goat serum and 0.3% Triton-X 100 in PBS) and incubated with primary antibodies in the same solution overnight at 4 °C. After washing, the sections were incubated with HSP60 antibody (sc-1052, Santa Cruz, Paso Robles, CA, USA) and TOM20 antibody (sc-11415, Santa Cruz), followed by Alexa Fluor 488 donkey anti-rabbit IgG (H + L) secondary antibody (Invitrogen, Carlsbad, CA, USA) and Alexa Fluor 594 donkey anti-goat IgG (H + L) secondary antibody (Invitrogen). To assess the recruitment of Pink-1 and Parkin to mitochondria, we used confocal microscopy and a pinhole setting of 0.25 AU to optimize signal detection. Pink-1 and Parkin were individually tagged with an Alexa Fluor 488 donkey anti-rabbit IgG (H + L) secondary antibody (Invitrogen), and mitochondria were visualized using TOM20 with an Alexa Fluor 594 donkey anti-goat IgG (H + L) secondary antibody (Invitrogen). Finally, the sections were counterstained with 4′,6-diamidino-2-phenylindole (DAPI) in mounting media (Invitrogen). Images were captured using a STED Super-Resolution microscope (Leica, Wetzlar, Germany).

### Assessment of activities of enzymes involved in oxidative phosphorylation

We used standard protocols to assess the activities of enzymes involved in oxidative phosphorylation in skeletal muscle tissues [54]. Muscle tissues were embedded in Tissue-Tek O.C.T. Compound (Sakura Finetek, CA, USA) and frozen in liquid nitrogen. Sections were cut from embedded muscles using a cryostat microtome. We then assayed for NADH, SDH, and cytochrome oxidase (COX) activities. The images were captured using an Olympus DP72 CCD device attached to an Olympus BX51 microscope (Tokyo, Japan).

### TEM examination

For EM, the skeletal muscle tissues were perfused with 8% formaldehyde and 2.5% glutaraldehyde in PBS. Both chemicals were procured from Sigma-Aldrich (St. Louis, MO, USA). We then post-fixed, processed, and imaged the muscle using established methods. Images were captured using a transmission electron microscope (H7650, Hitachi). We used ImageJ software to analyze the EM images and determine the mitochondrial population and size. The counting and area analysis functions in ImageJ were utilized, following an approach similar to that described by other researchers [55]. For area measurement, the mitochondria were circled using the least absolute shrinkage and selection operator tool. We then calculated the areas of the circles and converted these values into their actual values using a scale bar.

### Protein analysis

Skeletal muscles from mHSP60 (60H) mice were homogenized in radioimmunoprecipitation assay buffer (Amresco, OH, USA) with a protease inhibitor cocktail (Roche, Basel, Switzerland). Lysates were collected and quantified using a Pierce BCA Protein Assay Kit (Thermo Fisher Scientific, Waltham, MA, USA). Total proteins extracted from skeletal muscles were separated on 4−15% Mini-Protean TGX^TM^ Gels (BioRad, Hercules, CA, USA). For BN-PAGE, mitochondrial lysates (15 µg) were separated using the Native PAGE Novex Bis-Tris Gel System (Invitrogen).

The proteins were transferred to pure Protran Whatman nitrocellulose transfer and immobilization membranes (Sigma-Aldrich). These were reacted with antisera against HSP60 (sc-1052, Santa Cruz), TOM20 (sc-11415, Santa Cruz), MFN2 (GTX31923, Genetex, Irvine, CA), OPA1 (GTX48589, Genetex), NDUFB8 (ab110242, Abcam, Cambridge, UK), ATP5A (ab14748, Abcam), UQCRC2 (ab14745, Abcam), SDHB (ab14714, Abcam), MTCO1 (ab14705, Abcam), Pink-1 (GTX107851, Genetex), β-actin (MAB1501, Millipore), Parkin (GTX39745, Genetex), Bcl-2 (GTX100064, Genetex), Bcl-XL (GTX105661, Genetex), Bax (GTX109683, Genetex), BAK (GTX32463, Genetex), Caspase 8 (GTX31284, Genetex), PARP (GTX100573, Genetex), Beclin 1 (GTX133555, Genetex), ubiquitins (3933, Cell Signaling, MA), LC3B (2775, Cell Signaling), p62/SQSTM1 (P0067, Sigma-Aldrich), and LAMP1 (ab25245, Abcam). Immunolabeled proteins were revealed using ECL Plus (Millipore).

### Mitochondria isolation and proteomics analysis

Mouse limb skeletal mitochondria were isolated at 4 °C under sterile conditions using a Mitochondria Isolation Kit for Tissue (89801, Thermo Scientific, Waltham, IL, USA).

Pooled mitochondria from the muscle tissues of three mice were used for protein analysis. The Pierce™ BCA protein assay kit (Thermo Scientific) was used to determine protein concentrations. One-dimensional SDS–PAGE, in-gel protein digestion, nano-LC–MS/MS, database searching, and bioinformatics analysis of mitochondrial proteins from skeletal muscle tissue were performed following previously described standard protocols [56]. An in-house software program (Matrix Science, London, UK) was used to parse and summarize the Mascot search output files. Peptide identification was considered significant if the ion score was equal to or higher than the homology score (or the identity score, if no homology score was available). False-positive proteins were identified by searching the MS/MS spectra against the reversed sequence database. A false discovery rate of 1% was applied, requiring at least two peptides as significant protein hits.

### Measurement of mitochondrial DNA copy number

Genomic DNA was purified using phenol/chloroform extraction and ethanol precipitation. We determined the amount of mtDNA per nuclear genome using specific primer pairs (mtDNA: 5′-CCTATCACCCTTGCCATCAT-3′/5′-GAGGCTGTTGCTTGTG TGAC-3′; nuclear DNA, *Pecam* gene on chromosome 6: 5′-ATGGAAAGCCTGCCATCAT G-3′/5′-TCCTTGTTGTTCAGCATCAC-3′). We carried out quantification of relative copy number differences using both analyses of the difference in threshold amplification between mtDNA and nuclear DNA (ΔCt) and analysis with a standard curve of a reference template.

### Myoblast isolation and seahorse stress test

60H mice were euthanized using CO_2_. Their myoblasts were then isolated and digested using an adapted version of a published protocol [57]. The muscles were digested in a collagenase solution for 1 h at 37 °C, then centrifuged at 260×*g* for 5 min to remove the supernatant. The remaining pellets were resuspended in F-10-based primary myoblast growth medium (Thermo Fisher Scientific) and transferred to a collagen-coated culture dish. Myoblasts were cultured at 37 °C with 5% CO_2_, and the medium was replaced every 72 h.

The bioenergetics of mHSP60 myoblasts were studied using an XF24e Extracellular Flux Analyzer (Seahorse Bioscience, North Billerica, MA). Cells were kept at 37 °C in a CO_2_-free environment for 1 h before the measurement. We measured the basal OCR (mitochondrial respiration) and ECAR (lactic acid production or glycolysis). After four basal assay cycles, oligomycin (1 µM) was introduced to inhibit ATP synthase and estimate the respiration used for ATP synthesis. After three more assay cycles, carbonyl cyanide-4-(trifluoromethoxy) phenylhydrazone (1 µM) was added to stimulate maximal respiration in mitochondria. After another three assay cycles, rotenone (4 µM) and antimycin A (2 μM) were added to identify the nonmitochondrial respiratory rate, which was then subtracted from all other rates. To determine ECARs from glycolysis, 2-deoxy-glucose (100 mM; XF Cell Mito Glycolysis Test Kit, Seahorse Bioscience) was added, and measurements were taken. The ECARs were finally converted into proton production rates by considering the buffer capacity.

### Statistical analyses

To assess the differences between multiple groups, a one-way analysis of variance (ANOVA) was performed using SPSS v28.0. Prior to ANOVA, assumptions of normality and homogeneity of variance were evaluated using the Shapiro–Wilk and Levene’s tests, respectively. Data are presented as mean ± standard deviation, and *P* < 0.05 was considered statistically significant.

## Supplementary information

Original Western Blot

## Data Availability

This study did not generate or analyze any new data. Therefore, data sharing is not applicable to this article.
