# Supplementary figures and images for: Aberrant mitochondrial hsp60 expression affects mitochondria homeostasis and results in muscle dystrophy and premature death
Source: Cell Death Dis. 2026 Jan 8;17(1):9. doi: 10.1038/s41419-025-08260-1 (PMC12783612; doi:10.1038/s41419-025-08260-1)

Figure 1

B

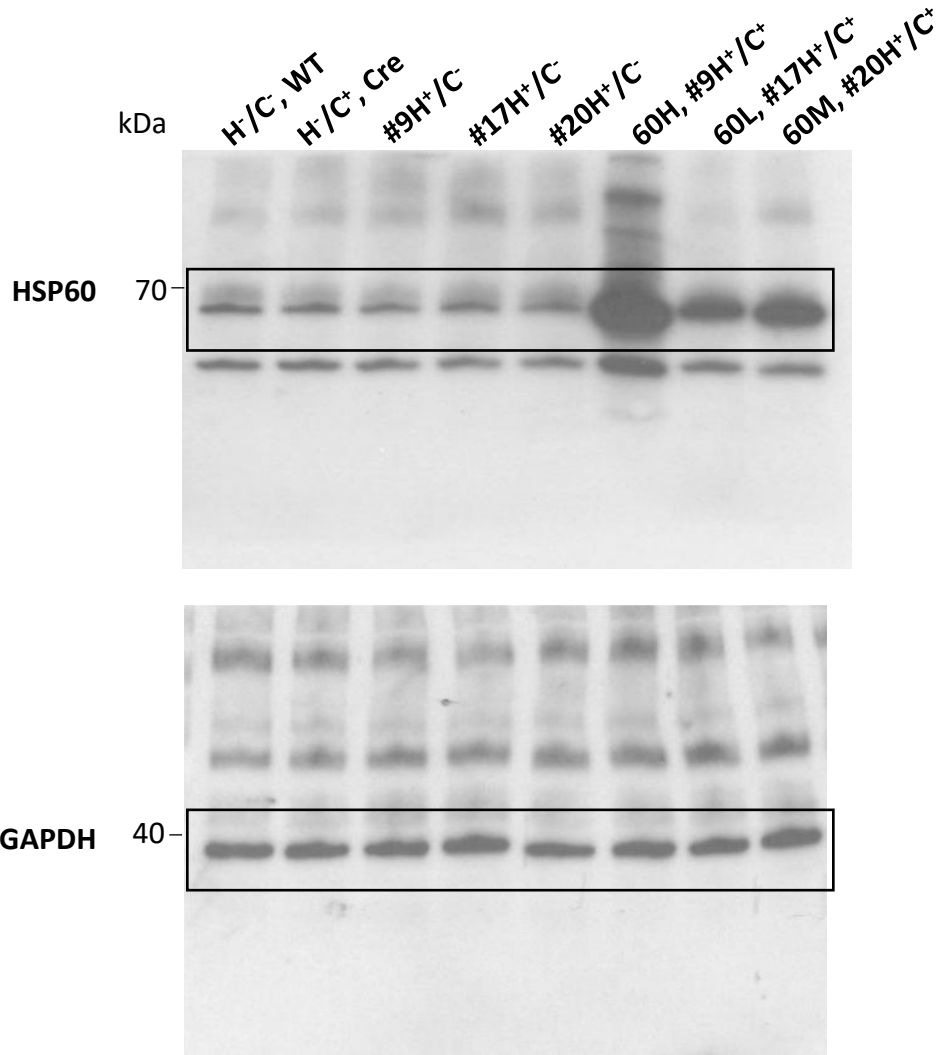

Figure 4

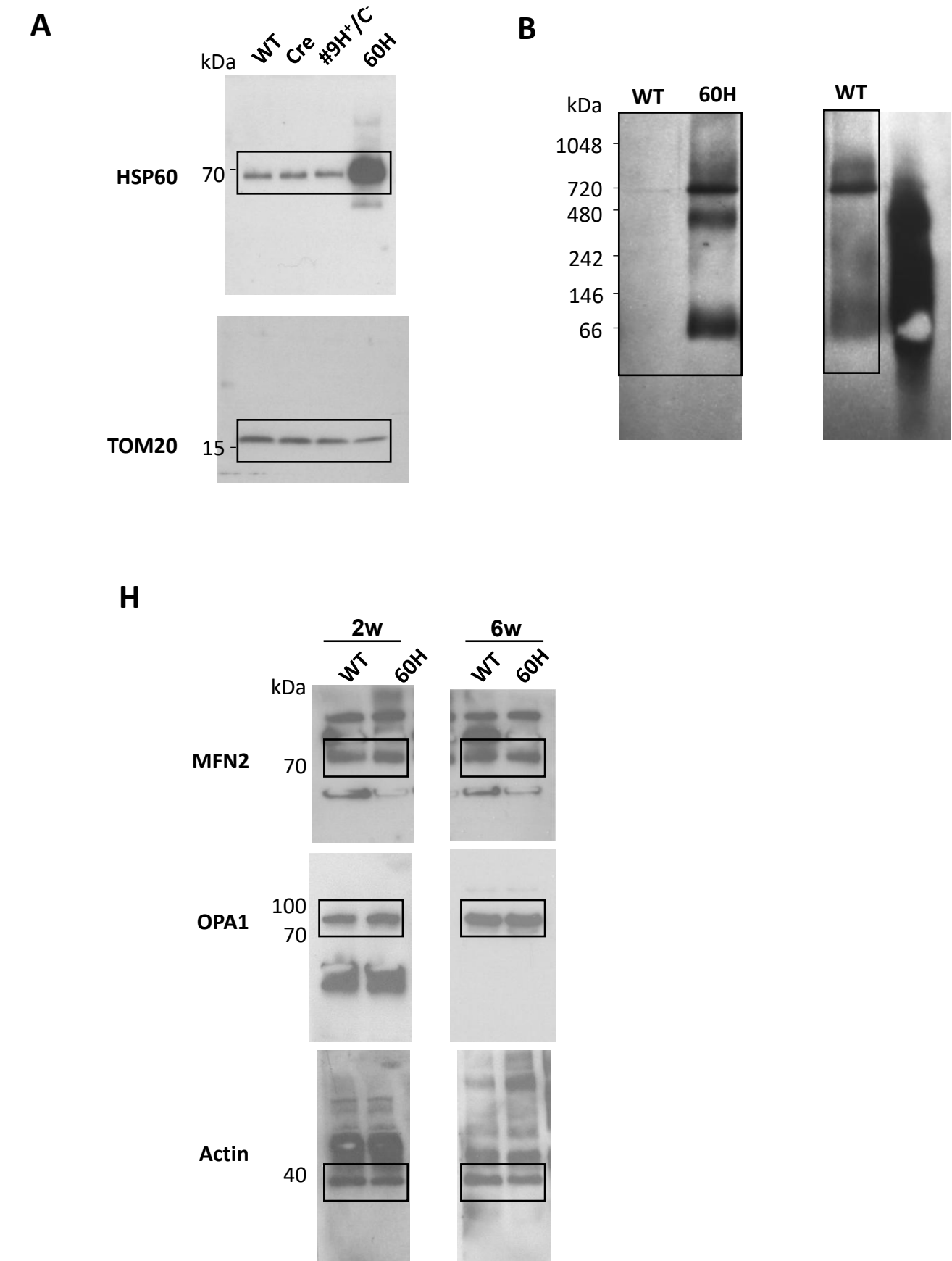

Figure 5

C

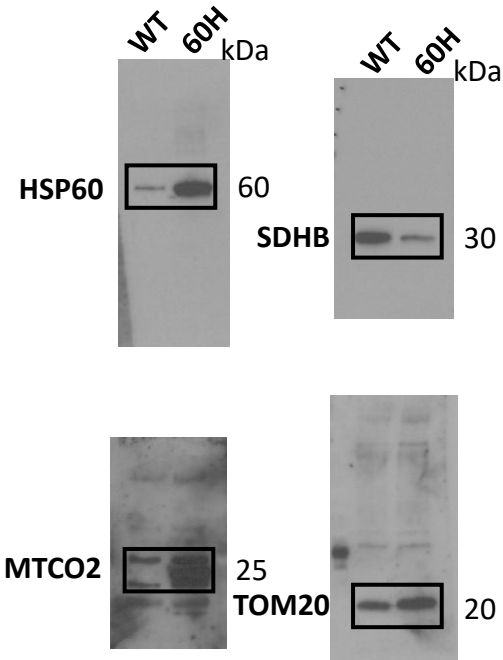

D

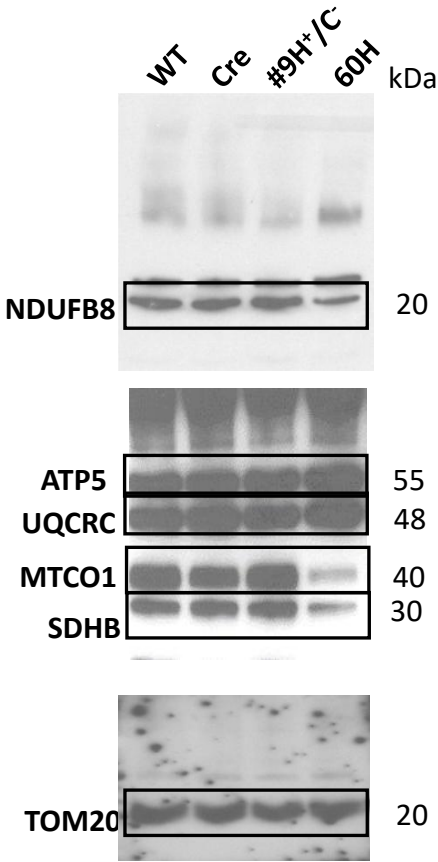

E

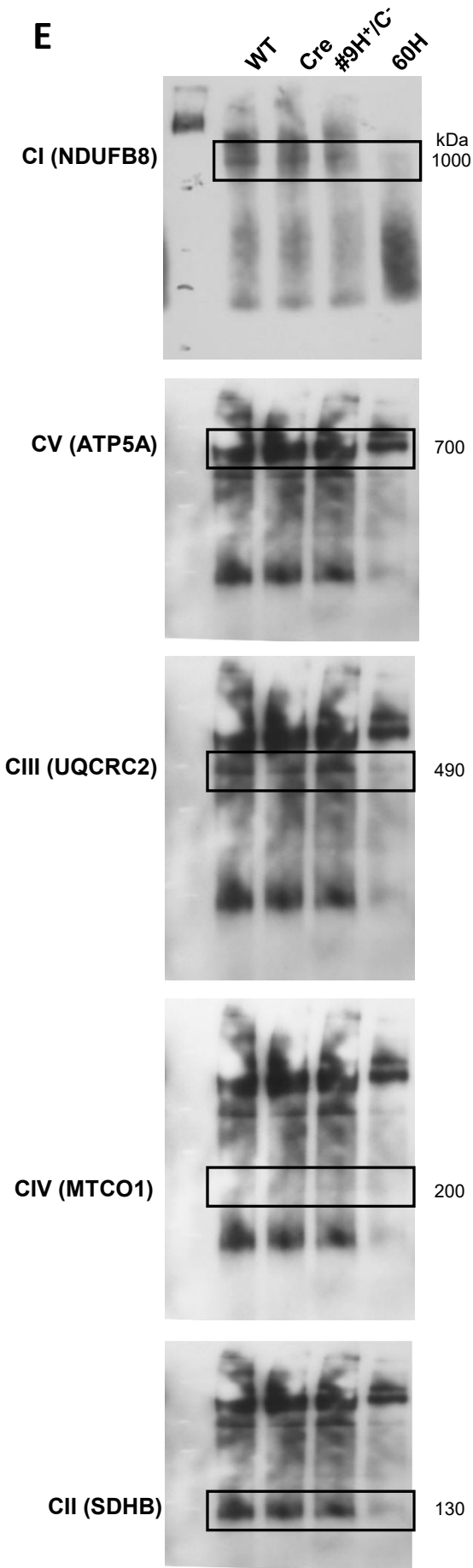

Figure 6

A

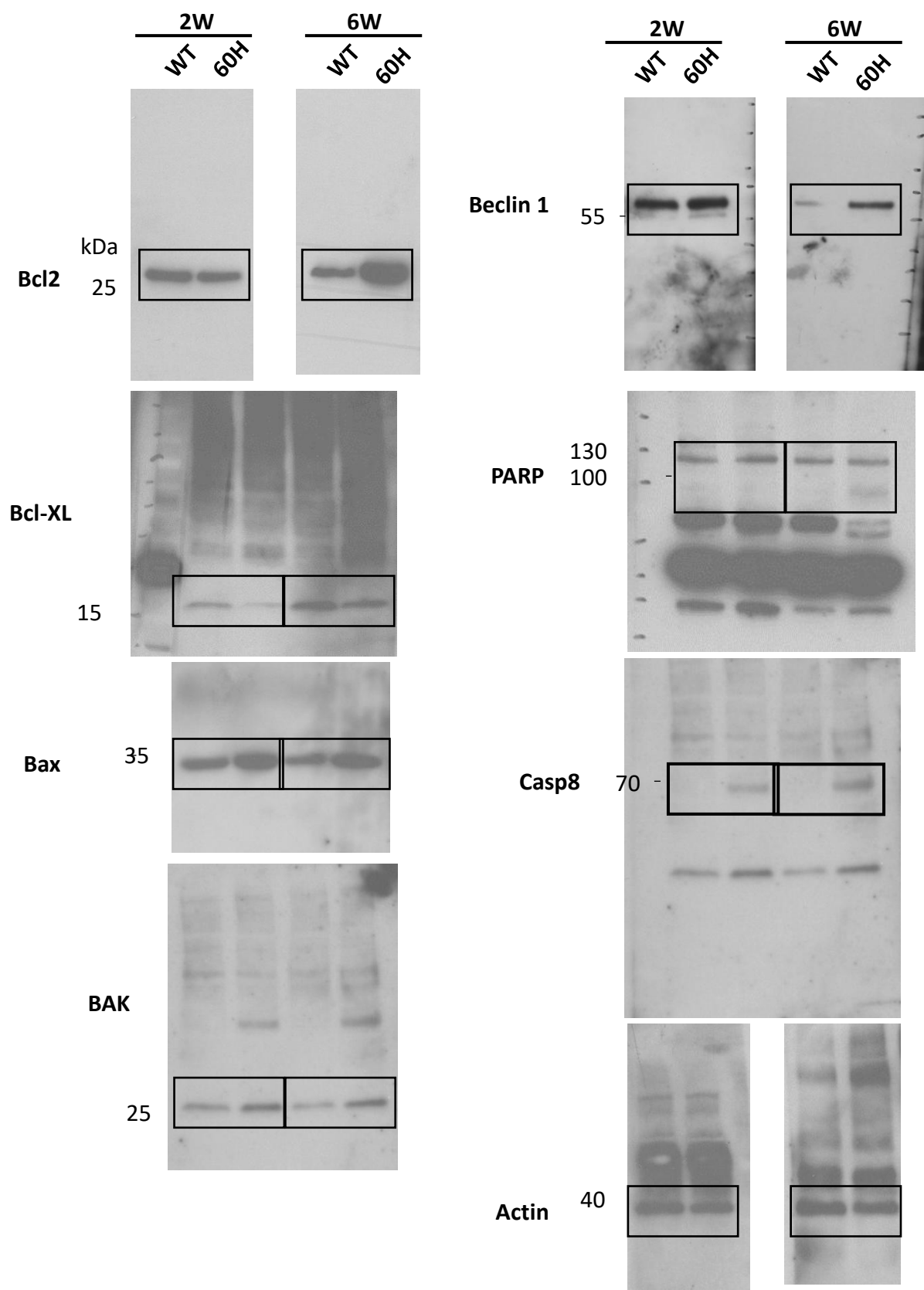

Figure 6

C

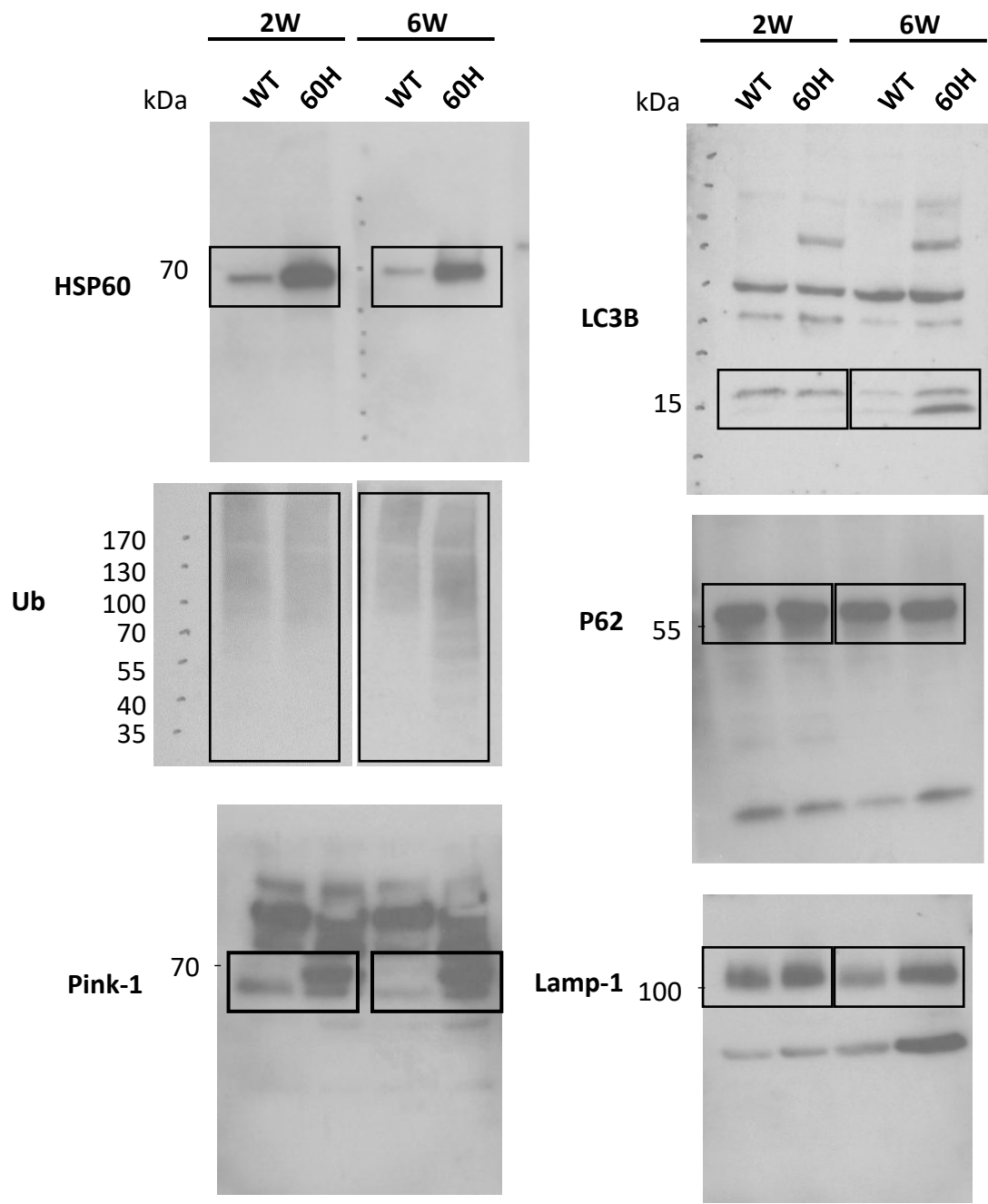

Figure 6

D

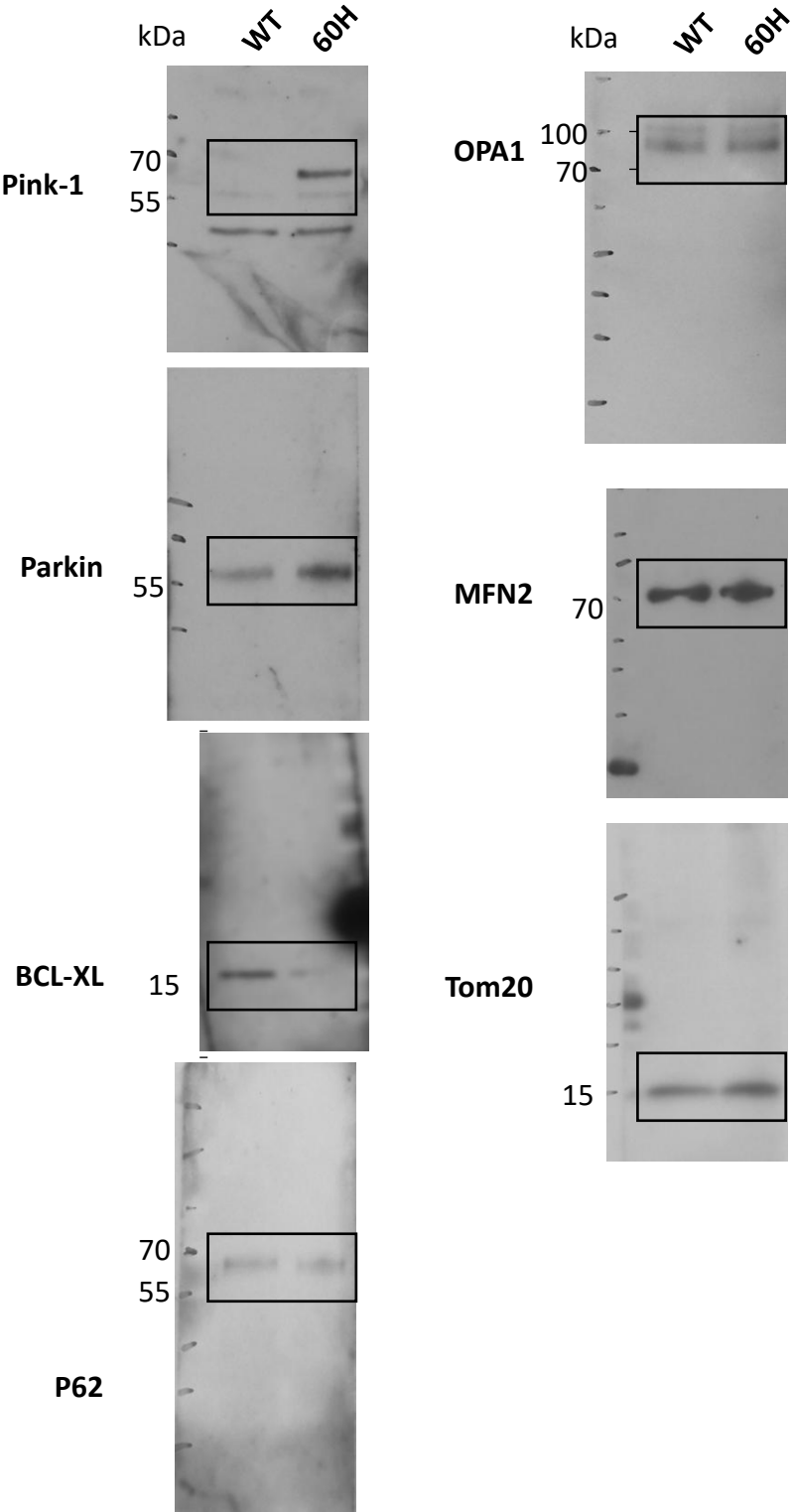

Supplement: Supplementary file 1 — Original Western Blot [file 41419_2025_8260_MOESM1_ESM.pdf]
